# Diverse roles of macrophage polarization in aortic aneurysm: destruction and repair

**DOI:** 10.1186/s12967-018-1731-0

**Published:** 2018-12-13

**Authors:** Zhao Cheng, Yang-zhao Zhou, Yin Wu, Qi-ying Wu, Xiao-bo Liao, Xian-ming Fu, Xin-min Zhou

**Affiliations:** 1 Department of Hematology, Institute of Molecular Hematology, The Second Xiang-ya Hospital, Central South University, Changsha, Hunan People’s Republic of China; 2 Department of Cardiovascular Surgery, The Second Xiang-ya Hospital, Central South University, Changsha, Hunan People’s Republic of China

**Keywords:** Macrophage polarization, Aortic aneurysm, Inflammation

## Abstract

Aortic aneurysm (AA) is defined as an enlargement of the aorta greater than 1.5 times its normal size. Early diagnosis of AA is challenging and mortality of AA is high. Curative pharmacological treatments for AA are still lacking, highlighting the need for better understanding of the underlying mechanisms of AA progression. Accumulating studies have proven that the polarization state of circulating monocyte-derived macrophages plays a crucial role in regulating the development of AA. Distinct macrophage subtypes display different functions. Several studies targeting macrophage polarization during AA formation and progression showed potential treatment effects. In this review, we focus on the recent advances of research on macrophage polarization in the progression of AA and propose that targeting macrophage polarization could hold great promise for preventing and treating AA.

## Introduction

Aortic aneurysm (AA) is a dilatation of the aorta and is associated with severe complications, such as aortic rupture. Although significant progresses have been made in both open and endovascular surgery, curative pharmacological treatments for this condition, especially for inoperative small AA (diameter less than 5.5 cm) are still lacking [1–3]. The underlying mechanisms involved in the formation and progression of AA have not been fully elucidated but are increasingly recognized to involve chronic inflammatory response, extracellular matrix (ECM) degradation, and matrix metalloproteinase (MMP) upregulation. Various types of inflammatory cells, including dendritic cells, B cells, T cells, neutrophils, mast cells, and macrophages, have been identified in both human and experimental AA tissues and have been shown to be associated with increased levels of proinflammatory cytokines and diverse proteases that contribute to AA development [4–9]. Of them, macrophages have been demonstrated to play a critical role in the formation and progression of AA. The two major macrophage phenotypes are known as classically-activated M1 macrophages (proinflammatory phenotype) and alternative activated M2 macrophages (anti-inflammatory phenotype) [10–12]. The main characteristic of M1 macrophages is the production of proteolytic enzymes and pro-inflammatory cytokines, such as tumor necrosis factor (TNF), Interleukin 6 (IL-6), IL-12, IL-1β, nitric oxide synthase 2 (iNOS), Chemokine (C-X-C motif) ligand 9 (CXCL9) and the CXCL10. In contrast, M2 macrophages participate in the anti-inflammatory response through secretion of factors such as IL-10 or transforming growth factor-β (TGF-β) and are involved in ECM remodeling and tissue repair [10, 13]. Recent findings have shown distinct macrophage subsets present in human and experimental murine AA tissues and are known to have different functions in the progression of AA. In this review, we provide current knowledge of the roles of distinct macrophage subsets on the development of AA, and particularly highlight their potential translational applications.

## The current treatments for aortic aneurysm

AA is an asymptomatic but life-threatening aortic disease and constitutes the 13th most common cause of death in the U.S. In developing countries such as China, as the population ages and dietary pattern changes the morbidity of AA is gradually increasing. Along with the diagnostic imaging technology progresses, the diagnostic rate of AA is also increasing [14]. In the current guidelines for AA treatment, for AA of diameter ≥ 5.5 cm, surgical resection, artificial vascular replacement or endoluminal stent repair are recommended. On the other hand, for small AA of diameter < 5.5 cm, the aortic dilation rate is slower, and the risk of aortic rupture is reduced; thus, traditional surgical treatment or endoluminal stent repair do not show obvious benefits. For patients with small AA, periodical imaging examinations to monitor the AA diameter are recommended; once the AA diameter reaches 5.5 cm or the dilatation rate of AA is higher than 0.5 cm per year, optional surgical repair or endoluminal stent repair would be considered. In summary, all current treatments for AA dependent on mechanical intervention. However, when most AAs are detected, they are below the threshold for repair, leading to a significant observation period during which there is currently no non-surgical therapy to prevent or slow AA expansion [15], highlighting a real need for better understanding of the potential mechanisms involved in the pathogenesis of AA.

## Macrophage polarization

**Fig. 1:**
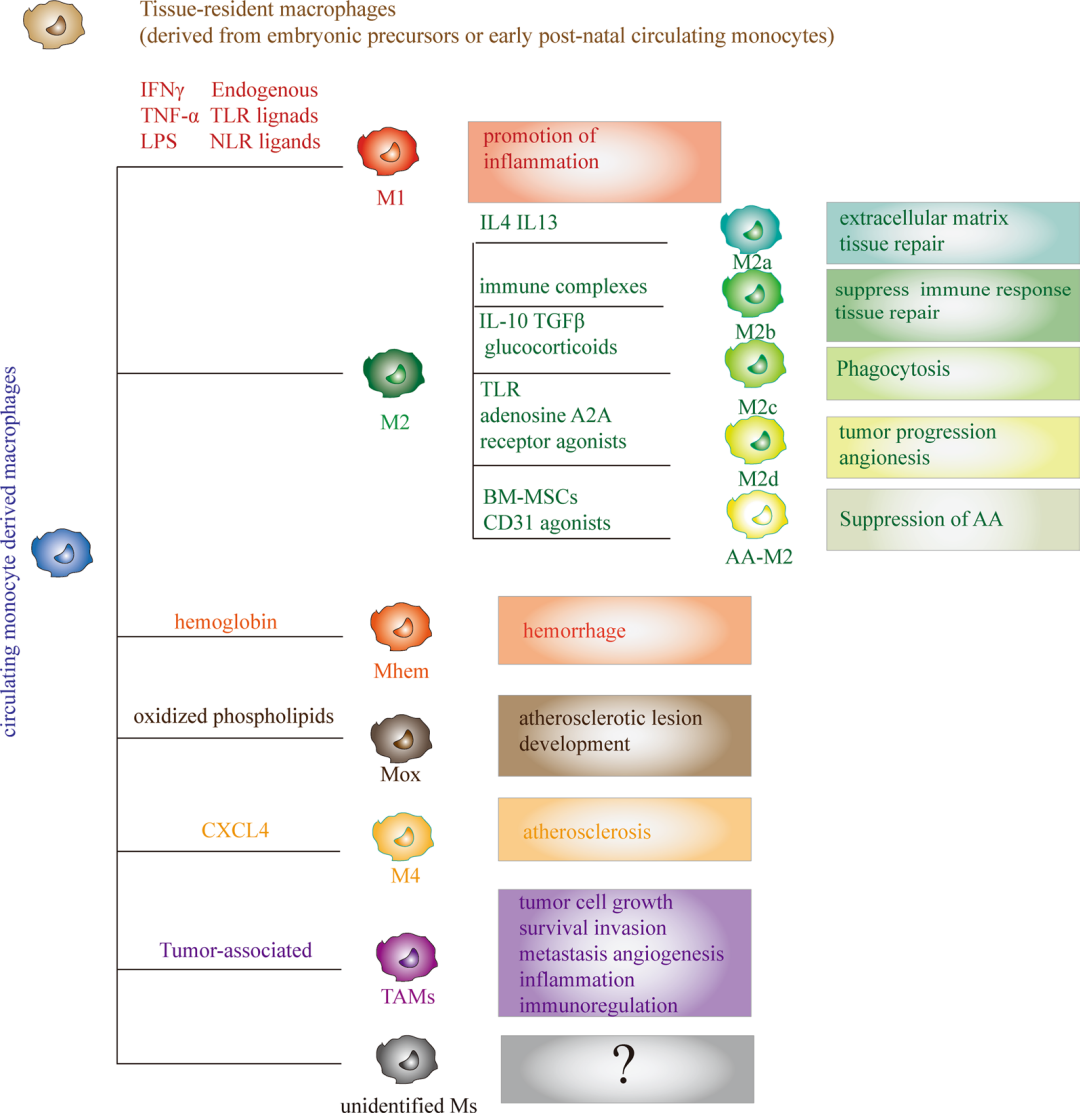
Characteristics of sub-populations of macrophages derived from circulating monocytes and its functions. Tissue-resident macrophages, derived from embryonic precursors or early post-natal circulating monocytes, play an important role in embryonic development. Macrophages derived from circulating monocytes can be categorized as various phenotypes. M1 macrophages can be induced from innate macrophages stimulated by IFNs, LPS, TNF-α, TLR ligands and NLR ligands. M1 macrophages function as pro-inflammatory macrophages. M2 macrophages are known as alternatively activated macrophages and can be subcategorized as M2a (stimulated by IL4, IL13; function is extracellular matrix tissue repair), M2b (stimulated by immune complexes; function is to suppress immune response and tissue repair), M2C (stimulated by IL-10, TGFβ, glucocorticoids; function is related to phagocytosis), M2d (stimulated by TLR, adenosine A2A, receptor agonists; function is tumor progression angiogenesis), AA-M2 (stimulated by BM-MSCs/CD31 agonists in AngII induced AA, function is suppression of AA). Mhem is associated with hemorrhage, which is induced by hemoglobin. Mox is generated by oxidized phospholipids. M4 macrophages are induced by chemokine ligand 4 (CXCL4), which is related to atherosclerosis. Tumor-associated macrophages (TAMs) are key players in the link between inflammation and cancer. There may be more unidentified subpopulation of macrophages that will be discovered in future studies. *IFNs* interferons, *AA* aortic aneurysm, *IL 4* interleukin-4, *IL13* interleukin-13, *TLR* Toll-like receptor, *NLR* NOD-like receptor, *AngII* angiotensin II, *TNF-α* tumor necrosis factor-α

Macrophages can be differentiated from circulating monocytes, bone marrow CD34^+^/CD86^+^ hematopoietic stem cells and CD25^+^/CD44^+^/FcR^+^ early T lymphocytes under appropriate conditions and can be distributed to various types of tissues [16, 17]. Most macrophages originate from circulating monocytes, which extravasate out of blood vessel and accumulate in the injured or inflammatory sites [18]. Macrophages are essential for the innate immune system. The main functions of macrophages are phagocytosis, antigen presentation, and cytokine secretion, which play important roles in inflammation, immune defense, tissue reconstruction, metabolism, and the maintenance of homeostasis [19, 20]. Under certain microenvironments or stimulation by certain factors, macrophages can be categorized as various phenotypes, such as “classically activated” M1 macrophages and “alternatively activated” M2 macrophages. M1 macrophages are effector cells of Th1 cells and can be induced from innate macrophages stimulated by interferons (IFNs), microbial lipopolysaccharide (LPS), or other cytokines, such as tumor necrosis factor (TNF), and macrophage colony-stimulating factor (M-CSF). These factors induce local pro-inflammatory microenvironments, which lead to the expression of CD68+, CD86+, and MHCII+ on macrophages and induce the overexpression of nicotinamide adenine dinucleotide phosphate(NADPH)-peroxidase and iNOS as well as the production of pro-inflammatory cytokines to perform efficient antigen presentation and eliminate antigens. Once M1 macrophages are consistently induced and activated, they may cause tissue damage. The phenotype of M2 macrophages is characterized as CD68+ and CD163+, and they can be induced by Th2 cytokines [10, 21, 22]. M2 macrophages can be sub-classified as M2a (stimulated by IL4 or IL13), M2b (stimulated by immune complexes in combination with IL-1β or LPS), M2c (stimulated by IL-10, transforming growth factor-β [TGF-β] or glucocorticoids) and M2d (stimulated by TLR and adenosine A2A receptor agonists). Recently, new phenotypes of macrophages were described to result from additional stimuli under certain microenvironments. These include hemorrhage-associated macrophages called Mhem (induced by hemoglobin) [23], macrophages stimulated with oxidized phospholipids (Mox) [24], and M4 macrophages induced by chemokine ligand 4 [25], tumor-associated macrophages (TAMs) etc. The currently available data indicate that macrophage polarization is a multifactorial process in which a large number of factors can be involved, producing different activation scenarios (Fig. 1). Once a macrophage adopts a phenotype, it retains the ability to continue changing in response to new environmental influences. The reversibility of polarization, also called functional adaptability, has critical therapeutic value, especially in diseases where the M1/M2 imbalance has a pathogenic role such as autoimmune diseases or chronic inflammation-related diseases. Key transcription factors are clearly associated with macrophage polarization, such as the signal transducer and activator of transcription family (STATs), peroxisome proliferator-activated receptor (PPAR), cAMP response element binding protein (CREB)-CCAAT/enhancer binding protein (C/EBP), hypoxia-inducible factors (HIF), nuclear factor kappa B (NFκB) and interferon regulatory factors (IRF) [26, 27].

## Macrophage polarization in the development of aortic aneurysm

Previous studies have shown that the main pathological features of AA include inflammatory cells infiltration, apoptosis of vascular smooth muscle cells (VSMCs), Upregulation of MMPs, and ECM degeneration. Inflammatory cells such as macrophages can infiltrate into aortic tissue and secrete MMPs and pro-inflammatory factors to promote destruction of ECM and induce apoptosis of VSMCs, resulting in AA formation. Studies have demonstrated that aortic injuries could induce the migration of bone marrow-derived cells to injury sites, where they differentiate into macrophages or fibroblasts, to participate in the immune response, repair and reconstruction of aortic wall [16, 17]. This phenomenon suggests that macrophages originating from bone marrow cells can function to repair the injured aortic tissues.

Numerous recent studies have suggested that M1 and M2 polarization of macrophages may be involved in the aortic remodeling and development of AA [18]. Moore et al. [19] reported that hypertension in mice with angiotensin (Ang II) infusion is associated with accumulation of Ly6C^hi^ monocytes in the aortic tissues. These cells differentiate into M2 macrophages, which likely promote ECM remodeling, including collagen deposition and elastin loss. Using laser capture microdissection (LCM) and immunohistochemistry, Boytard et al. [20] observed the distribution of subtypes of macrophages in human AA tissues. They found that M1 macrophages (CD68+MR−) were predominant in the adventitia, while M2 macrophages (CD68+MR+) were predominant in the intraluminal thrombus. They also found that stabilin 1, involved in the uptake and degradation of unwanted molecules, was overexpressed in M2 (CD68+MR+) macrophages, which suggested that M1 and M2 macrophages may play different roles in AA development. Hans et al. [21] reported that Notch1 haploinsufficiency prevents the influx of proinflammatory macrophages (M1) at the aneurysmal site by causing defects in macrophage migration and proliferation. Decreased levels of Notch1 protects against the formation of AA by preventing macrophage recruitment and attenuating the inflammatory response in the aorta. Hasan et al. [22] found that M1 and M2 macrophages are present in equal proportions in unruptured aneurysms. However, there is an increase in M1 macrophages and mast cells in ruptured aneurysms. A macrophage M1/M2 imbalance and upregulation of mast cells may play roles in the progression of cerebral aneurysms to rupture. Batra et al. [23] reported that IL (interleukin)-1β was differentially expressed in human plasma from patients with AA compared with matched atherosclerotic controls. Wang et al. [24] found that TNF-stimulated gene-6 (TSG-6) was elevated in both the plasma and aortic wall of patients with AA compared with healthy and risk-factor matched non-AA donors. Both IL-1β and TSG-6 are related with the regulation of macrophage polarization in AA. In our primary study, we observed that, at early stages of AngII induced AA in Apo E^−/−^ mice, M1 macrophages were increased along with secretion of several pro-inflammatory cytokines and chemokines. After AA formation, M2 macrophages markedly increased along with secretion of anti-inflammatory cytokines and chemokines.

**Fig. 2:**
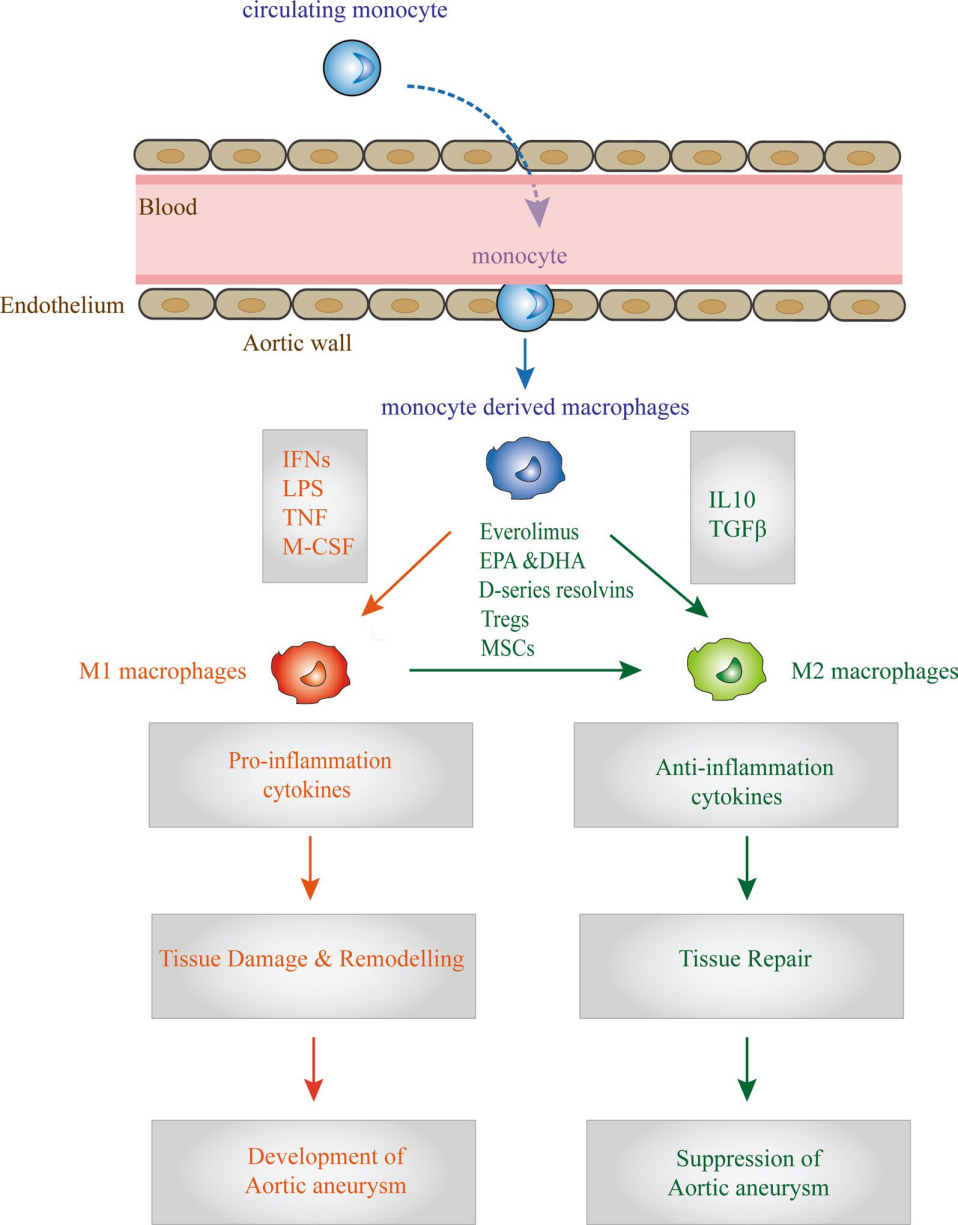
Macrophage polarization in AA and its functions. M1 and M2 polarization of macrophages derived from circulating monocytes were found to be closely related with the development and prognosis of AA. The chronic inflammatory microenvironments at the site of AA, such as the expression of IFNs, LPS, TNF and M-CSF, induce M1 macrophages, which function as pro-inflammatory macrophages; in response to inflammation, they can cause tissue damage related to the development of AA. On the other hand, M2 macrophages induced by IL-10 and TGFβ can function as anti-inflammatory macrophages, which play a key role in tissue remodeling. Some molecules or factors have been reported to induce the transformation of M1 macrophages into M2 macrophages, such as Everolimus, EPA & DHA, D-series resolvins, Tregs and MSCs. *AngII* angiotensin II, *AA* aortic aneurysm, *ApoE* apolipoprotein E, *MMP* matrix metalloproteinase, *Tregs* regulatory T cells, *BM-MSC* bone marrow derived mesenchymal stem cells, *IL-1β* interleukin-1β, *EPA* eicosapntemacnioc acid, *DHA* docosahexaenoic acid, *TGFβ* transforming growth facto, *M-CSF* macrophage colony-stimulating factor

Based on the above evidence, distinct macrophage subtypes are believed to regulate aortic remodeling after injury (Table 1). At the early stage of AA, M1 macrophages dominate at the site of injured aortic tissues function in inflammatory factors expression, proteolysis, and phagocytosis. At late stage, M2 macrophages accumulate preferentially and facilitate reparative processes such as ECM deposition and angiogenesis. Maintenance of an appropriate M1/M2 ratio is crucial for aortic tissue homeostasis (Fig. 2). Understanding the role of macrophage polarization in aortic remodeling may lead to the development of novel therapies for AA.

**Table 1:** Macrophage subtypes involved in aortic remodeling and aneurysm

Disease modle/objects	Pathway/key regulators	References
Ang II-infused ApoE^−/−^ mice	M2 macrophage accumulation in the aortic wall	Moore et al. [19]
Human aneurysmal infrarenal aortic wall	M1 macrophage was predominant in the adventitia while the M2 macrophages in the intraluminal thrombus/stabilin 1	Boytard et al. [20]
Ang II-infused ApoE^−/−^ mice	Notch1	Hans et al. [21]
Cerebral aneurysms	Macrophage M1/M2 imbalance and upregulation of mast cells	Hasan et al. [22]
Patients with AA	IL-1β was differentially expressed in human plasma with in patients with AA	Batra. et al. [23]
Patients with AA	NF-stimulated gene-6 (TSG-6) elevated in both the plasma and aortic wall of patients with AA	Wang et al. [24]

*AngII* angiotensin II, *IL-1β* interleukin-1β, *ApoE* apolipoprotein E, *AA* aortic aneurysm, *TSG-6* tumor necrosis factor-inducible gene 6

## Modulating macrophage function for treatment of aortic aneurysm

Macrophage polarization can regulate inflammation, thus affecting the development and prognosis of inflammation-related diseases. Recent studies have shown that using drugs or cell therapy modulating macrophage polarization could achieve therapeutic effects for AA [25–27]. Moran et al. [25] showed that systemic administration of the rapamycin inhibitor everolimus limits AA in the AngII-infused ApoE^−/−^ mouse model via suppressed development of bone marrow CCR2 monocytes, reduced egress of these cells into the circulation and diminished IFNγ/lipopolysaccharide-stimulated M1 polarization in bone marrow monocyte-differentiated macrophages. In Yoshihara et al. [26] study, the AA model was developed by AngII infusion in ApoE^−/−^ mice. AA formation and macrophage infiltration were significantly suppressed after Eicosapntemacnioc Acid (EPA) and Docosahexaenoic acid (DHA) administration. The expression of arginase 2, a marker of pro-inflammatory macrophages (M1), was significantly lower, and that of Ym1, a marker of anti-inflammatory macrophages (M2), was significantly higher after EPA and DHA administration. Pope et al. [27] reported that administration of D-series resolvins could attenuate murine AA formation by increasing M2 macrophage polarization and altering inflammatory cytokine expression. Dale et al. [28] showed that treatment of bone marrow-derived macrophages with Elastin-derived peptides (EDPs) could induce M1 macrophage polarization. Injection of M2-polarized macrophages reduced aortic dilation after aneurysm induction. EDPs promoted a pro-inflammatory environment in aortic tissues by inducing M1 polarization, and neutralization of EDPs attenuated aortic dilation. Andreata et al. [18] found that CD31 agonist P8RI induces the switching of M1macrophages to the reparative M2 phenotype and promotes the healing of experimental dissected aortas in Apo E^−/−^ mice with Ang II infusion. Meng et al. [29] reported that adoptive transfer of Tregs dose-dependently prevented AngII-induced AA in ApoE^−/−^ mice. One of the underlying mechanisms was that Tregs downregulated M1-related genes and upregulated M2-related genes, thus regulating macrophage polarization. Our group found that bone marrow derived mesenchymal stem cells (BM-MSCs) could regress the formation of AA by regulating macrophage polarization to restore the M1/M2 ratio and to reduce inflammation at the site of AA [30–32].

Taken together, these studies mentioned above demonstrate that the M1/M2 macrophage ratio plays a fundamental role in AA formation, development, and progression. Macrophage function can be stimulated or inhibited to improve healing and repair. The augmentation of aortic tissue repair by reducing M1 phenotype macrophage infiltration or promoting polarization to a reparative M2 phenotype seem be attractive therapeutic strategies for AA (Table 2).

**Table 2:** Macrophages polarization in the treatment of aortic aneurysm

Agents	Experiment model	Pathway/key regulators	Treatment effect	References
Everolimus	AngII-induced AA in ApoE^−/−^ mice	Bone marrow development of Ly6C + CCR2 + (inflammatory) monocytes	Decrease aortic dilatation	Moran et al. [25]
EDPs	CaCl_2_ induced AA in C57BL/6 mice	Modulating M1/M2 macrophage polarization	Promote AA	Dale et al. [28]
D-series resolvins	Elastase-induced AA in C57/B6 mice AngII-infused ApoE^−/−^ mice	Increasing M2 macrophage polarization	Decrease in MMPs Attenuated AA formation and progression	Pope et al. [27]
Tregs	AngII-induced AA in ApoE^−/−^ mice	Downregulated macrophage type 1–related genes and upregulated macrophage type 2–related genes	Declined proinflammatory cytokine expression and MMP-2 and MMP-9 levels and enhanced anti-inflammatory cytokine expression	Meng et al. [29]
BM-MSCs	AngII-induced AA in ApoE^−/−^ mice	BM-MSC inhibited infiltration of M1 macrophages and preserved the construction of elastin	Decrease vascular inflammation Prevent AA expansion	Yamawaki-Ogata et al. [31]
IL-1β TNF-α	CaCl_2_ induced AA in C57BL/6 mice	TNF-α deletion but not IL-1β deletion, inhibited M1 macrophage polarization	Infusion of M1 polarized TNF-α^−/−^ macrophages inhibited aortic diameter growth	Batra et al. [23]
CD31 agonist P8RI	AngII-induced AA in ApoE^−/−^ mice	CD31 signaling promotes the switching of proinflammatory macrophages to the reparative phenotype	Promoting the resolution of intramural hematoma and the production of collagen in dissected aortas	Andreata et al. [18]
EPA and DHA	AngII-induced AA in ApoE^−/−^ mice	Promote macrophage polarization toward the M2 phenotype	Inhibited aortic inflammation, degeneration and macrophage infiltration	Yoshihara et al. [26]

*AngII* angiotensin II, *AA* aortic aneurysm, *ApoE* apolipoprotein E, *MMP* matrix metalloproteinase, *Tregs* regulatory T cells, *BM-MSC* bone marrow derived mesenchymal stem cells, *IL-1β* interleukin-1β, *TNF-α* tumor necrosis factor-α, *EPA* eicosapntemacnioc acid, *DHA* docosahexaenoic acid

## Summary and prospects

In the present review, we summarized recently published studies on the roles of macrophage polarization in the aortic remodeling and development of AA. We highlighted the functions of macrophage subsets, which are complex, and could be either destructive or reparative during AA development. M1 macrophages accumulate in the aortic wall and dominate the cellular milieu and mainly clear cellular debris at early stage of AA. Thereafter, M1 macrophages secrete inflammatory cytokines that affect the consequent phases of aortic remodeling and initiate aortic tissue repair coordinated by M2 macrophages. The prolonged effects of M1 macrophages extend the destructive effects of inflammatory responses and cause expansion of AA. More recently, studies targeting macrophage differentiation towards the M2 phenotype have been shown to promote the resolution of aortic inflammation and slow AA progression. Modulation of macrophage subset polarization is believed to be an attractive strategy to prevent the progression of AA. However, macrophage polarization phenotypes are not always mutually exclusive, and it still remains unsolved whether some functional subsets represent real distinct populations. Additionally, the molecular and cellular mechanisms underlying regulation of macrophage polarization during AA development are complex and multifactorial. Further studies with more animal models and patients will be needed to be conducted to determine the precise roles of macrophage polarization in AA in order to develop effective treatment and prevention strategies.
